# Corneal blindness: Prevention, treatment and rehabilitation

**Published:** 2009-12

**Authors:** Matthew J Burton

**Affiliations:** Senior Lecturer, International Centre for Eye Health, London School of Hygiene and Tropical Medicine, Keppel Street, London WC1E 7HT, UK. Honorary Consultant, Kilimanjaro Christian Medical Centre, Moshi, Tanzania.

**Figure:**
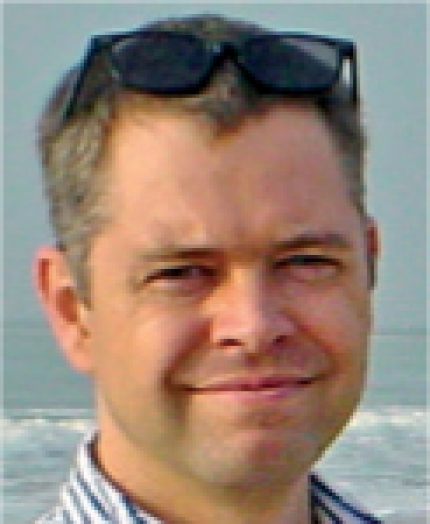


## The patient

Mr Massae (not his real name) is a forty-eight year old farmer who recently presented at a tertiary ophthalmology unit in Tanzania with a three-week long history of pain, purulent discharge, and loss of vision in the left eye. Several days after the onset, he had received treatment from his local health centre (chloroamphenicol drops), but his eye continued to get worse. At presentation, he had a large corneal ulcer with infiltration and a hypopyon. A filamentous fungus was cultured from microbiology specimens. He was treated with intensive topical antifungal (econazole) and anti-bacterial (ciprofloxacin) drops, a topical cycloplegic (atropine), and an oral antifungal medication (itraconazole). A small corneal perforation developed which plugged with iris and then sealed. The infection gradually responded to prolonged antifungal therapy, leaving a dense scar and small eccentric pupil (Figure 1, over page). Four years earlier, Mr Massae had lost sight in his right eye due to severe suppurative keratitis following a minor corneal abrasion from a maize leaf; this caused dense scarring of his right cornea (Figure 2, over page). Mr Massae is now blind.

**Figure:**
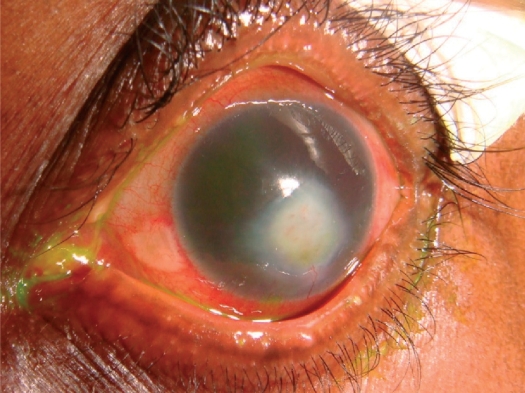
Figure 1. Mr Massae's left eye, after several weeks of treatment

Matthew Burton

**Figure:**
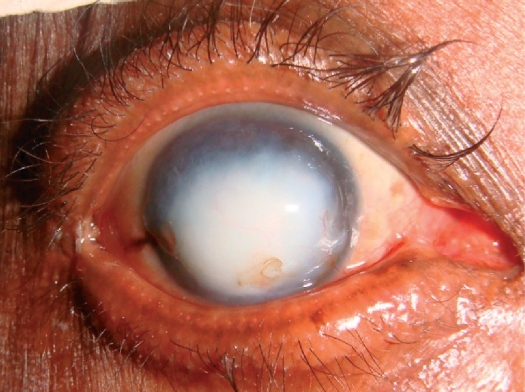
Figure 2. Mr Massae's right eye

Matthew Burton

## The burden

Unfortunately, Mr Massae's story is not unique. Blindness from corneal disease is a major ophthalmic public health problem. According to the most recent WHO global data on the causes of blindness (2002), ‘corneal opacities’ affected 1.9 million people (5.1% of the total number of bind people). If other conditions causing blindness through corneal pathology are included, such as trachoma, vitamin A deficiency, ophthalmia neonatorum, and onchocerciasis, the number would be significantly higher. Moreover, there are probably many tens of millions more who are blind in one eye from corneal disease.

**Figure:**
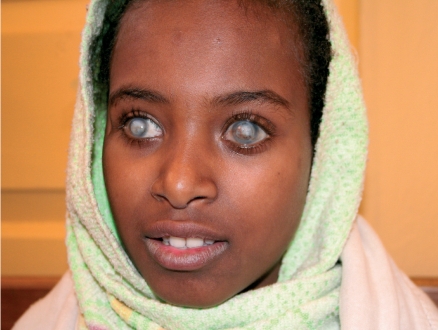
Corneal blindness often affects people at a young age, such as this twelve year old girl who is blind from vitamin A deficiency. She has had a penetrating corneal graft in her left eye; this has unfortunately failed. ETHIOPIA

Lance Bellers

The burden of corneal blindness on the individual and the wider community can be huge, particularly as it tends to affect people at a younger age than other blinding conditions such as cataract and glaucoma. It also disproportionately affects poor rural communities, because of the increased risk of eye injuries from contaminated objects such as plant material, limited access to treatment, and higher prevalence of communicable diseases such as trachoma and onchocerciasis. Mr Massae illustrates the burden from corneal disease: he is currently unable to farm his land and provide food for his family.

## The causes

There are many different conditions which can damage the structure and shape of the cornea leading to visual impairment and blindness. These include infectious, nutritional, inflammatory, inherited, iatrogenic (doctor-caused), and degenerative conditions (see box opposite). Disease patterns vary in different environments. Overall, in low- and middle-income countries, infectious keratitis tends to be the most common problem. However, other conditions, such as trachoma or onchocerciasis, may dominate in some areas.

## Controlling corneal blindness

There are three important elements to addressing corneal blindness: prevention, treatment, and rehabilitation. In this issue of the *Community Eye Health Journal*, you will find articles addressing aspects of each of these. In Mr Massae's case, we can see how all three elements are needed, as well as some of the challenges in their implementation.

**Figure:**
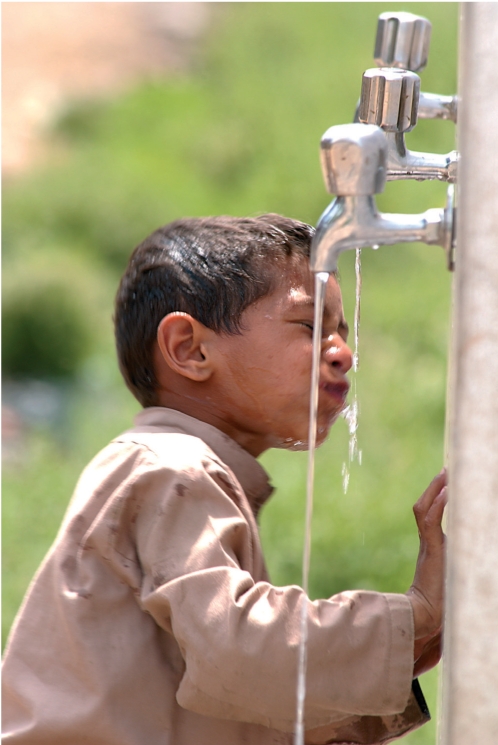
Trachoma is one of the most common causes of corneal blindness. Improvement in water supply facilitates facial cleanliness, one of the four components of the SAFE strategy for trachoma control. PAKISTAN

Jamshyd Masud/Sightsavers

## Prevention

Some blinding corneal conditions are very difficult to treat once established; however, they can be prevented by specific public health interventions (see page 36).

- **Xerophthalmia**, which is caused by vitamin A deficiency and sometimes precipitated by measles, accounts for more than half the new cases of childhood blindness each year. In addition to blindness, these young children are at increased risk of death. Prevention is key: vitamin A supplementation, measles vaccination, and nutritional advice have led to a marked reduction in this condition.
- **Trachoma**, caused by recurrent infection with *Chlamydia trachomatis*, causes blinding corneal opacification through the traumatic effect of entropion/trichiasis and possibly secondary bacterial infection. Once established, trachomatous corneal opacification is difficult to treat: the results of corneal grafting are often disappointing, in part due to a dry and damaged ocular surface. Blinding trachoma can be prevented through the full implementation of the SAFE Strategy (Surgery for trichiasis, Antibiotics for infection, Facial cleanliness and Environmental improvement to control transmission).
- **Onchocerciasis (river blindness)** leads to blindness through an inflammatory response to the microfilaria of *Onchocerca volvulus* in the retina and the cornea. Control programmes have been very effective in preventing blindness through the mass distribution of ivermectin and measures to control the *Simulium* fly.
- **Traumatic corneal abrasion** is a common event and is the major risk factor for microbial keratitis in low- and middle-income countries. Simple topical antibiotic prophylaxis for a few days while the epithelium heals can protect the eye from developing potentially blinding infection. Mr Massae lost the sight in his right eye after an abrasion caused by vegetable matter. It is possible that early antibiotic prophylaxis could have prevented this.

## Treatment

In most low- and middle-income countries, microbial keratitis is the most common acute blinding corneal problem requiring treatment. There is often a history of minor trauma. If appropriate antibiotic prophylaxis is not started soon after the injury, infection can become established. In temperate climates, most infections are bacterial. In contrast, in tropical regions, fungal keratitis is more frequent and may account for about half the cases.

The treatment of microbial keratitis is discussed in detail in the articles on pages 39–41.

Several problems make it difficult to deliver effective treatment for microbial keratitis in a low- and middle-income country setting. These problems need to be addressed by eye care programmes in order to reduce the risk of blindness from microbial keratitis and include:

- **Delayed presentation.** There may be many days or even weeks between the onset of symptoms and the presentation of the patient at an appropriate health facility. This delay is often catastrophic, allowing time for deep-seated infection to develop and extensive corneal damage to occur. Timely presentation may be promoted through health education and training of staff at primary health facilities to recognise and refer patients with established microbial keratitis.
- **Traditional medication.** This may sometimes be used by the patient before presentation and can make the problem more severe through the harmful effect of toxic compounds and infection with additional microorganisms.
- **Microbiology.** It may not be possible to obtain a microbiological diagnosis or information about the sensitivity of the organism. This can lead to the use of ineffective treatment and is particularly a problem when fungal keratitis is missed. The development of a basic microbiological service with gram staining of slides can help to identify some cases of fungal infection. Blindness control programmes need to know which organisms commonly cause microbial keratitis in their population as well as their pattern of antibiotic resistance so that appropriate drugs can be supplied to health facilities.
- **Inadequate treatment.** The patient may not receive effective treatment. This can occur for several reasons: appropriate antibacterial or antifungal drops may not be available, the microorganism may be resistant to the medication, or the drops may not be given with sufficient intensity. Some of these problems can be overcome with the development of locally appropriate treatment protocols.

Mr Massae's case illustrates some of these issues. It was several weeks before he reached an ophthalmology unit and received treatment, which contributed to the severity of the case. It was very helpful in his management to have a microbiological diagnosis as it guided the choice and duration of treatment.

## Rehabilitation

Mr Massae is now blind. However, his left eye has perception of light and has the potential to see better; to be rehabilitated. It may be possible to offer him some improvement in vision with a pupilloplasty. In addition, as the left corneal scar does not involve the superior cornea, a rotational auto-graft, in which an eccentric corneal button is cut and rotated to bring the clear superior cornea into the centre, may help. However, a penetrating corneal graft (transplant) would probably offer him the best quality of vision.

In many low- and middle-income countries, options for visual rehabilitation from corneal disease are limited as it usually requires the services of an ophthalmologist with sub-specialty training in corneal surgery, equipped to perform the surgery and with access to donated corneas from an eye bank.

In this issue, there is an article on corneal grafting (page 44) which discusses the indications for corneal grafting, the outcomes for different conditions, and some of the potential complications. The following article focuses on eye banking (page 46) and addresses some of the specific challenges involved in running an eye bank service and finding donors.

Without rehabilitation services, Mr Massae and several million people like him are destined to a life without sight. Without the implementation of the public health and treatment interventions outlined above, many more will be at risk of joining them.

**Causes of corneal blindness**

**Infectious**
- Bacterial keratitis
- Fungal keratitis
- Viral keratitis
- Trachoma
- Onchocerciasis
- Leprosy
- Ophthalmia neonatorum

**Nutritional**
- Vitamin A deficiency (xerophthalmia)

**Inflammatory**
- Mooren's ulcer
- Steven's Johnson Syndrome

**Inherited**
- Corneal stromal dystophies
- Fuch's endothelial dystrophy

**Degenerative**
- Keratoconus

**Trauma**
- Corneal abrasion predisposing to microbial keratitis
- Penetrating trauma
- Chemical injury

**Doctor-caused (iatrogenic)**
- Pseudophakic bullous keratopathy

